# MIEN1 drives breast tumor cell migration by regulating cytoskeletal-focal adhesion dynamics

**DOI:** 10.18632/oncotarget.10798

**Published:** 2016-07-23

**Authors:** Marilyne Kpetemey, Pankaj Chaudhary, Timothy Van Treuren, Jamboor K. Vishwanatha

**Affiliations:** ^1^ Department of Molecular and Medical Genetics, Institute for Cancer Research, and the Texas Center for Health Disparities, University of North Texas Health Science Center, Fort Worth, TX 76107, USA

**Keywords:** MIEN1, actin polymerization, focal adhesion kinase, cofilin, migration

## Abstract

Migration and invasion enhancer 1 (MIEN1) is an important regulator of cell migration and invasion. MIEN1 overexpression represents an oncogenic event that promotes tumor cell dissemination and metastasis. The underlying mechanism by which MIEN1 regulates migration and invasion has yet to be deciphered. Here, we demonstrate that MIEN1 acts as a cytoskeletal-signaling adapter protein to drive breast cancer cell migration. MIEN1 localization is concentrated underneath the actin-enriched protrusive structures of the migrating breast cancer cells. Depletion of MIEN1 led to the loss of actin-protrusive structures whereas the over-expression of MIEN1 resulted in rich and thick membrane extensions. Knockdown of MIEN1 also decreased the cell-substratum adhesion, suggesting a role for MIEN1 in actin cytoskeletal dynamics. Our results show that MIEN1 supports the transition of G-actin to F-actin polymerization and stabilizes F-actin polymers. Additionally, MIEN1 promotes cellular adhesion and actin dynamics by inducing phosphorylation of FAK at Tyr-925 and reducing phosphorylation of cofilin at Ser-3, which results in breast cancer cell migration. Collectively, our data show that MIEN1 plays an essential role in maintaining the plasticity of the dynamic membrane-associated actin cytoskeleton, which leads to an increase in cell motility. Hence, targeting MIEN1 might represent a promising means to prevent breast tumor metastasis.

## INTRODUCTION

Solid tumors like breast cancers that have developed distant metastases as a result of tumor cell dissemination are often associated with poor survival and unfavorable prognosis [1–3]. The acquisition of migratory ability is required for the spread of tumor cells to distant organs. Migration is a phenomenon that requires the molecular coordination of actin-rich membrane protrusions, adhesion, and contractility to achieve directed cell migration and subsequent invasion [4–6]. These processes are regulated by numerous metastasis promoting and suppressing proteins [3, 7]. Thus, identifying novel metastatic proteins and their action mechanisms may provide new insights into the pathogenesis and management of tumor metastasis.

Migration and invasion enhancer 1 (MIEN1), previously known as C35, C17orf37, MGC14832, RDX12, ORB3 and XTP4, was first reported to be highly expressed in human breast tumors and its expression was shown to persist from early events in tumorigenesis to late stages of the disease [8]. MIEN1 gene is located in the human chromosomal region 17q12-21 next to the ERBB2 or Her-2/Neu oncogene in a tail-to tail arrangement [9, 10]. MIEN1 is frequently amplified along the neighboring genes, *ERBB2* and *GRB7* in variety of tumors including breast cancer [11, 12]. MIEN1 is post-translationally modified by geranyl-geranyl transferase-I (GGTase-I), which adds an isoprenyl group to the carboxyl-terminal CVIL motif of the protein [8, 13]. Prenylated MIEN1 associates with the inner leaflet of the plasma membrane and mediates signaling through the Akt/NF-kB axis to influence the expression of extracellular matrix-degrading proteases and angiogenic factors such as such as matrix metalloproteinase (MMP)-9 and urokinase-type plasminogen activator (uPA) and vascular endothelial growth factor (VEGF) [13, 14]. In addition to the prenylation and redox-active motifs, MIEN1 also contains a canonical immunoreceptor tyrosine-based activation motif (ITAM) reported to be associated with epithelial to mesenchymal transition (EMT)-mediated invasion in breast cancer and essential to MIEN1 induced motility [15, 16]. Using pre-clinical animal models, MIEN1 was shown to enhance the metastatic ability of tumor cells by promoting their dissemination and colonization to distant sites [13, 17].

Previous studies have attributed a role to MIEN1 in tumor cell migration by inducing filopodia formation and subsequent dissemination of cancer cells to distant organs [13–15, 17–19]. However, the molecular mechanisms underlying the effects elicited by MIEN1 on breast tumor cell migration remain elusive. The present studies elucidate the role of MIEN1 in the regulation of actin cytoskeletal dynamics to influence cell motility. We found MIEN1 localizes to focal adhesions and stress fibers in the lamellum, a region that plays a major role in actin-rich membrane protrusions. Consequently, modulation of MIEN1 expression significantly affected actin-rich membrane protrusions and cell-substratum interactions. Our results demonstrate for the first time that MIEN1 enhances F-actin polymerization through the cofilin and focal adhesion kinase (FAK) pathways. The present study suggests that MIEN1 might be a key cytoskeletal signaling adaptor protein that regulates actin dynamics and cell adhesion during motility in breast cancer.

## RESULTS

### Localization of MIEN1 during cell migration

**Figure 1:**
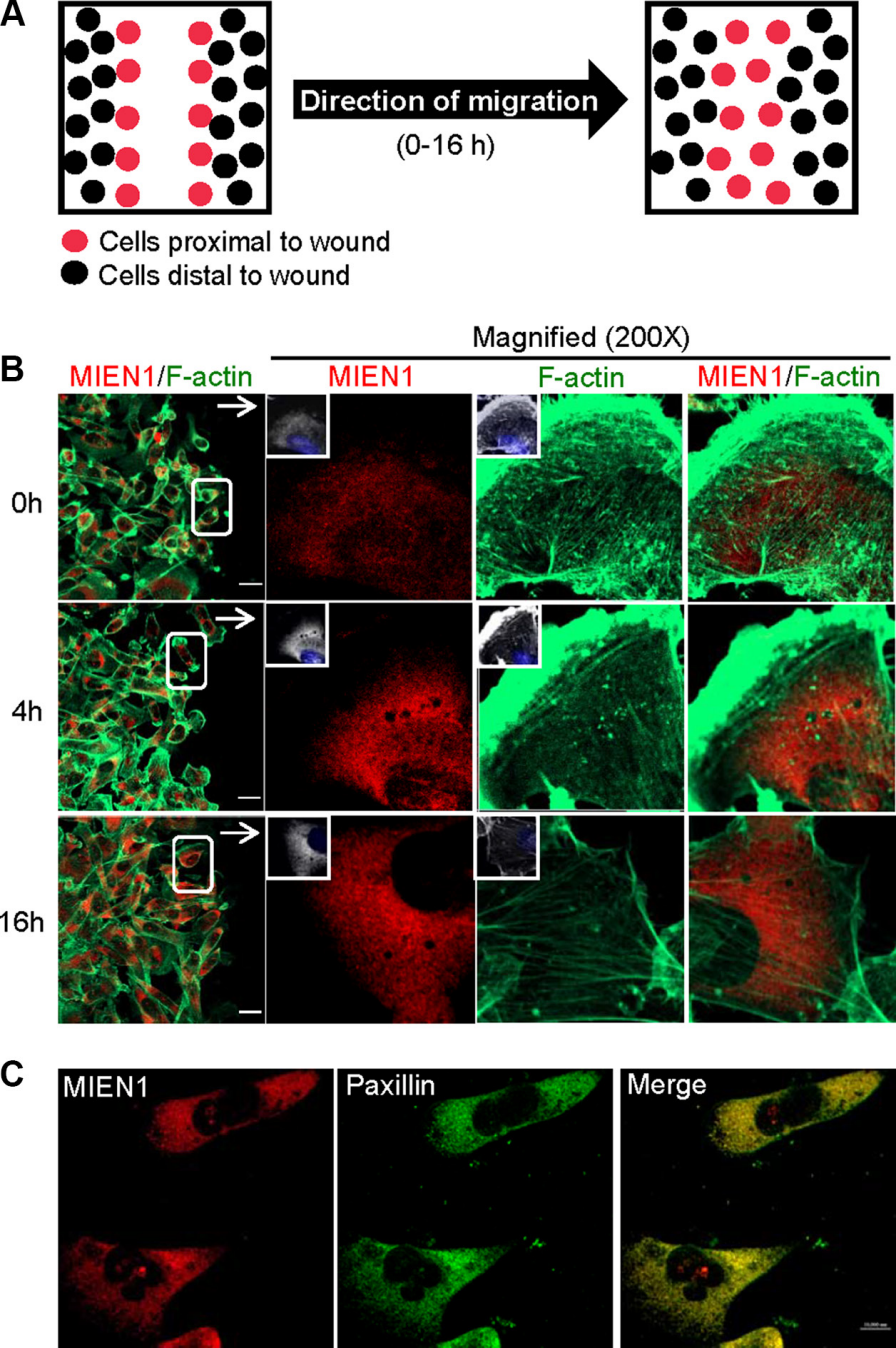
MIEN1 localization in migrating cells (**A**) Schematic representation of wound-healing assay used. (**B**) Monolayer cultures of MDA-MB-231 cells were wounded, fixed at various time points and stained for MIEN1 (Red), F-actin (Green) and nuclei (blue). (**C**) Immunofluorescence staining of MIEN1 (Red) and paxillin (Green) in MDA-MB-231 cells. Yellow dots represent the colocalization of green and red colors, which indicates that MIEN1 is colocalized with paxillin.

Previous studies have shown that over-expression of MIEN1 induces filopodia formation which results in increased migratory behavior in both *in vitro* and *in vivo* models [13, 17]. It has also been demonstrated that post-translational modification by isoprenylation targets MIEN1 to the plasma membrane, an association critical to its functions [13, 18]. In an effort to determine the role of MIEN1 in increased breast cancer cell motility, we first examined the intracellular localization of endogenous MIEN1 in relation to actin filaments by immunostaining (Figure 1). A wound was induced to stimulate migration and only cells migrating to fill the wound were analyzed (Figure 1A). Immunofluorescence of MDA-MB-231 cells with an anti-MIEN1 antibody demonstrated that in stationary cells (0 h), MIEN1 is concentrated in the cytoplasm and in the perinuclear region as previously shown [13, 14, 17]. At various time points (4 h and 16 h) following wound induction, immunolocalization showed MIEN1 staining to be diffuse throughout observed cells (Figure 1B). Co-staining of MIEN1 and F-actin revealed no colocalization but rather showed prominent staining of MIEN1 lying underneath the actin-rich protrusive structures of the membrane. The leading edge of migrating cells is defined by two actin networks: the lamellipodium, characterized by a fast retrograde flow powered by F-actin polymerization, and the lamellum, which is a more stable network with slow retrograde flow that occupies a larger area and is associated with stress fibers and focal adhesions [20–22]. Thus, we tested the association of MIEN1 with paxillin, a component of focal adhesions in migrating cells [23, 24]. Co-staining with paxillin indicated that MIEN1 localized to focal adhesions in MDA-MB-231 (Figure 1C) and MCF10CA1a cells (Supplementary Figure S1). All together, these results clearly show that MIEN1 is concentrated in the cytoplasm of migrating cancer cells and localized to focal adhesions.

### MIEN1 is required for actin-driven membrane protrusions

**Figure 2:**
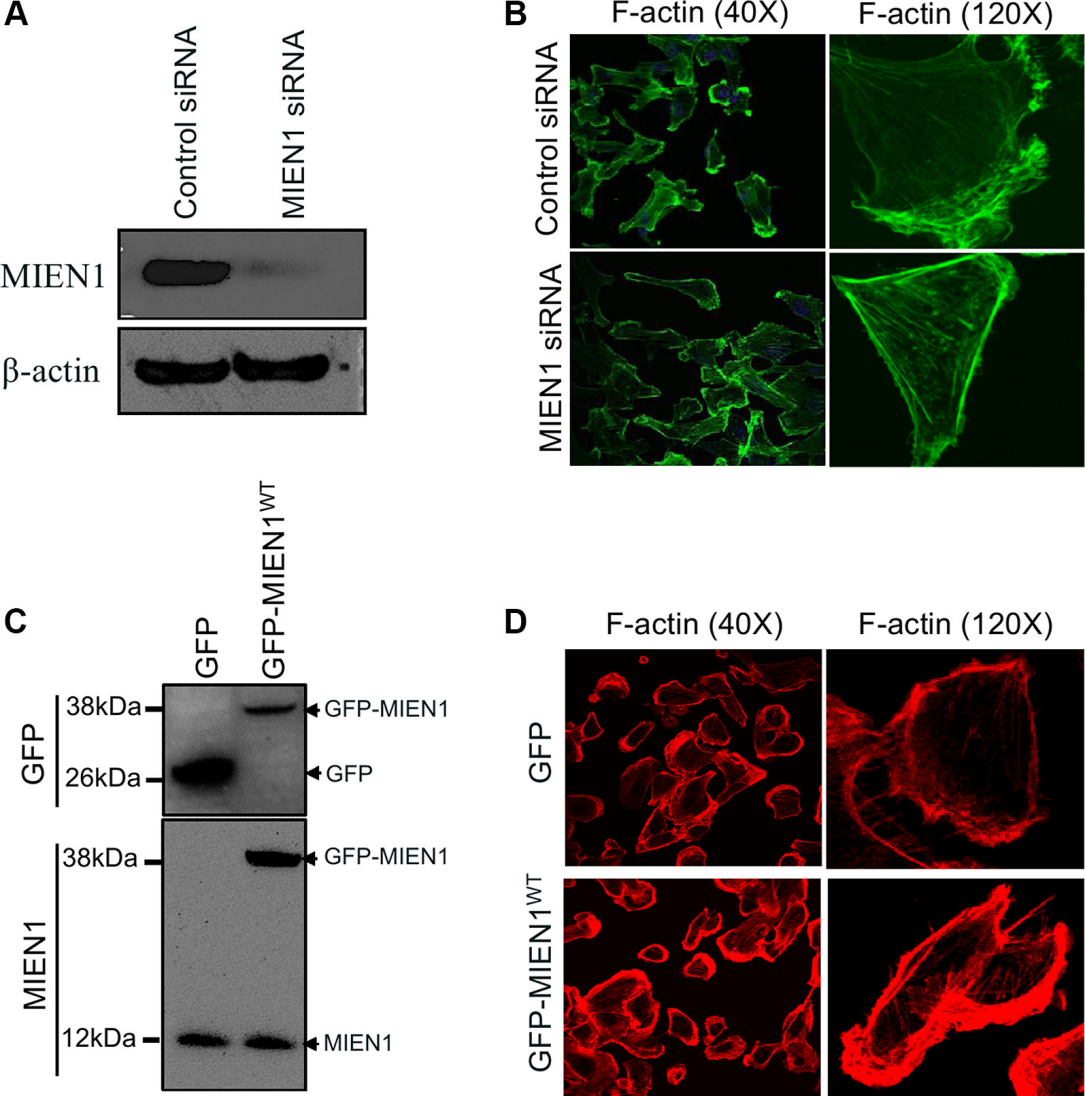
MIEN1 regulates actin-rich membranes protrusions during cell migration (**A**) MDA-MB-231 cells were treated with control and MIEN1 siRNA. Cell extracts were analyzed by Western blotting for MIEN1 expression at 72 h post-transfection. β-actin was used as loading control for total cell lysate. (**B**) Immunofluorescence staining was performed using Alexa 488-labeled phalloidin (F-actin; green) in control and MIEN1 siRNA treated MDA-MB-231 cells. (**C**) Western blot analysis shows MIEN1 expression upon GFP vector control or GFP-MIEN1 plasmid transfection in MDA-MB-231 cells. (**D**) MDA-MB-231 cells expressing the GFP vector control or GFP-MIEN1 plasmid constructs were fixed and stained with rhodamine conjugated phalloidin and examined for membrane protrusions.

Cell migration involves actin-rich protrusions directing adhesion and de-adhesion cycles followed by translocation of the cell body forward and retraction of the rear [25–28]. All of these events are governed by an intricate reorganization of the actin cytoskeleton [29, 30]. To examine whether the observed spatio-temporal localization of MIEN1 correlates with its role in motility, we examined the effects of MIEN1 depletion on actin-rich protrusions using short interfering RNA (siRNA) and confocal microscopy. Transfection of MDA-MB-231 cells with siRNA specific to MIEN1 resulted in ~90% reduction of MIEN1 at the protein level after 72 h of transfection when compared with control siRNA transfection (Figure 2A). After 72 h of siRNA transfection, subconfluent monolayers of cells were stimulated for migration by a scratch wound and were monitored for changes in morphology and actin based structures (Figure 2B). Confocal analysis showed that control siRNA treated cells had prominent lamellipodium ruffles and, within the cell body, stress fibers were seen in low abundance as illustrated by F-actin staining with Alexa-488 phalloidin. The most striking phenotype of cells depleted of MIEN1 through siRNA was the accumulation of thick F-actin stress fibers throughout the cytoplasm and the disappearance of membrane ruffles. Similarly in MCF10CA1a cells, the suppression of MIEN1 resulted in a flattened morphology accompanied by the loss of lamellipodium extensions and membrane ruffles (Supplementary Figure S2A and S2B). To confirm that the defects in cytoskeletal rearrangements were a direct consequence of MIEN1 depletion, we introduced GFP vector control and GFP-tagged MIEN1 in MDA-MB-231 and MCF10CA1a cells. In agreement with the previous observations, the over-expression of MIEN1 resulted in an increase in actin-rich membrane extensions in both MDA-MB-231 (Figure 2C and 2D) and MCF10CA1a cells (Supplementary Figure S2C and S2D) compared to the GFP vector control expressing cells. These results confirmed that decreased MIEN1 accumulation at the leading edge lamellum had a functional impact on the rearrangements of actin rich membrane protrusions during cell motility.

### MIEN1 is required for cell-substratum adhesion

**Figure 3:**
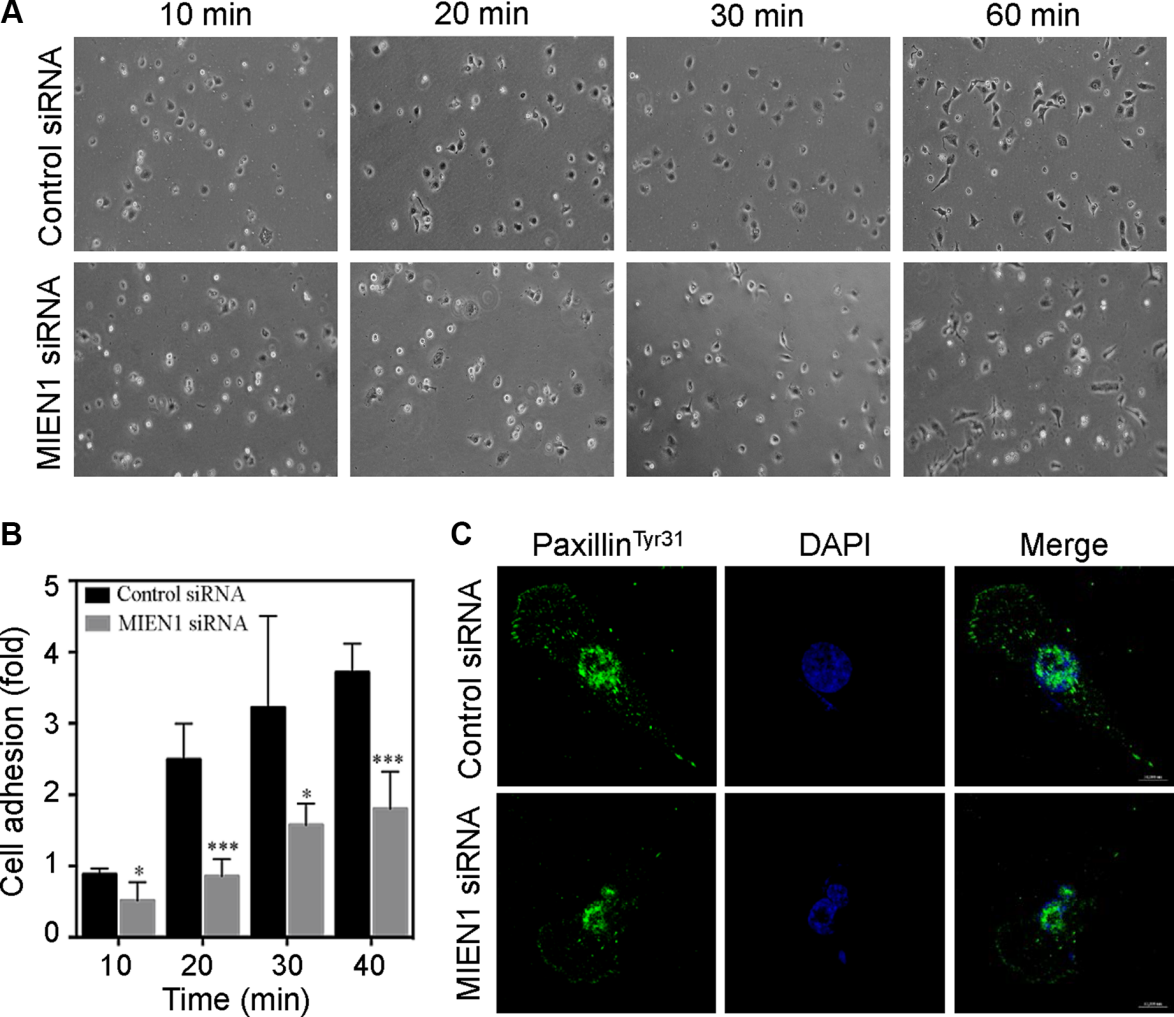
Depletion of MIEN1 affects focal complex turnover and cell adhesion (**A**) Phase imaging of control siRNA treated MDA-MD-231 cells plated on fibronectin-coated coverslips showed enhanced spreading and adhesion compared to MIEN1 knockdown cells. At the times indicated, phase-contrast micrographs were taken with a Nikon inverted microscope equipped with a 10X objective lens. (**B**) The data represent quantification of adherent cells from 5 random sites. The experiment was repeated twice. (*p < 0.05; ***p < 0.001 vs same time-point control). (**C**) MDA-MD-231 cells were cultured on fibronectin-coated coverslips and transfected with control or MIEN1 siRNA for 72 h. Coverslips were fixed and stained with paxillin^Tyr31^ (green). Nuclei were stained with DAPI (blue).

Membrane protrusions underlie cell–substratum interactions and direct cell locomotion [25, 27, 31]. MIEN1 localization to focal adhesions and its ability to regulate membrane extensions suggested that it is also involved in adhesion cycles. We then investigated the consequences of MIEN1 depletion on cell adhesion. To carefully compare the adhesion and spreading efficiency, we trypsinized control and MIEN1 siRNA transfected MDA-MB-231 cells at 72 hours post-transfection and washed them in serum-free media supplemented with trypsin soybean inhibitor. The cells were then resuspended in serum free media and plated on fibronectin coated cell culture dishes. After time-lapse imaging, we found that control siRNA treated cells initiated spreading within 20 min after plating, with an average of 100 cells per field attaining a flat morphology in 30 min (Figure 3A and 3B). At 60 min, nearly the majority of control cells appeared more extended and elongated with a flattened morphology. In contrast, the majority of MIEN1 depleted cells looked smaller with a rounded morphology after 30 min of plating and only few had spread by 60 min relative to the control cells. As shown in the graph (Figure 3B), our results clearly demonstrated that depletion of MIEN1 significantly reduced the cell-substratum adhesion in MDA-MB-231 cells. It has been reported that phosphorylation of paxillin (a cytoskeletal component that localizes to the focal adhesions at the ends of actin stress fibers) at tyrosine 31 is affected under changes in actin-driven cytoskeletal rearrangements and cell adhesion [32, 33]. Therefore, we examined the effect of MIEN1 depletion on tyrosine 31 phosphorylation of paxillin by confocal immunofluorescence analysis in MDA-MB-231 cells. In control cells, we found thick doted staining of phosphorylated paxillin present at the membrane and throughout the cell body whereas in MIEN1 depleted cells, there was a visible reduction in phosphorylated paxillin throughout the cell along with the disappearance of thick doted spots corresponding to focal adhesions of the cell (Figure 3C). Collectively, these results affirmed a role for MIEN1 in cell-substratum adhesion.

### MIEN1 participates in actin filament reorganization to promote cytoskeletal dynamics

**Figure 4:**
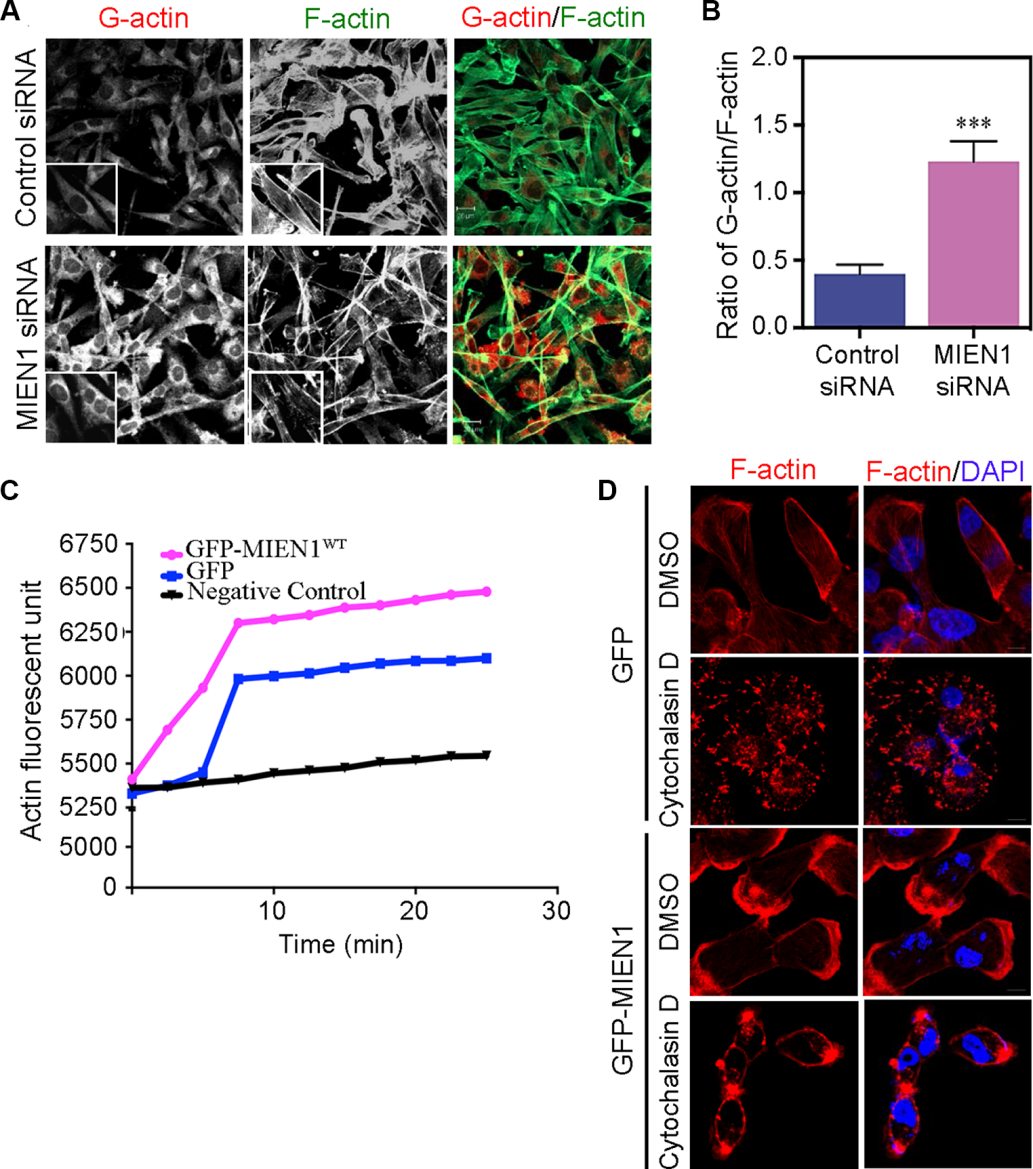
Loss of MIEN1 affects G-actin/F-actin contents and filaments reorganization (**A**) Expression of G-actin and F-actin in MDA-MB-231 cells treated with control or MIEN1 siRNA were analyzed by immunofluorescence staining with deoxyribonuclease I Alexa-594 conjugated (red) and phalloidin Alexa-488 conjugated (green), respectively. Representative immunofluorescence images are shown. (**B**) The quantification of G-actin/F-actin ratio intensities were evaluated from at least 10 fields per treatment. (***p < 0.001 vs control). (**C**) *In vitro* pyrene-labeled actin polymerization assay was performed in control or MIEN1 siRNA treated MDA-MB-231 cells. The reaction with only actin and buffer (black) served as the control. (**D**) MDA-MB-231 cells expressing GFP vector control or GFP-MIEN1 plasmid constructs were treated with DMSO and Cytochalasin D (1 μM) for 1 h. The cells were fixed, stained with rhodamine-conjugated phalloidin (red) and DAPI (blue).

Focal adhesions are multi-protein complexes that link the extracellular matrix to the actin cytoskeleton through stress fibers [28, 34]. A variety of actin binding proteins including side-binding proteins, capping proteins, and nucleators make up focal complexes [20, 28, 35]. Our previous studies showed that MIEN1 localizes along stress fibers to focal adhesions in the lamillum of migrating cells and promotes actin-driven membrane protrusions and cell- substratum adhesion. These results support the speculation that MIEN1 participates in the assembly of the actin stress fibers to focal complexes. To further test this hypothesis, we asked whether the monomeric globular (G)-actin-to-filamentous (F)-actin ratio can be affected by the depletion of MIEN1 in MDA-MB-231 cells. Immunofluorescence staining of G- and F-actin showed that MIEN1 depletion increased the amount of G-actin in these cells whereas the levels of F-actin decreased (Figure 4A). Fluorescence quantification of G-actin to F-actin ratio revealed a distinct shift toward the prevalence of F-actin in control cells compared with MIEN1 knockdown MDA-MB-231 cells suggesting that MIEN1 might be involved in early events of F-actin assembly (Figure 4B). To determine whether MIEN1 influences the assembly of actin monomers into filaments, we expressed GFP vector control and GFP-MIEN1 wild type in MDA-MB-231 cells and analyzed their activity in an *in vitro* F-actin polymerization assay (Figure 4C). The results showed that the slope of the actin polymerization curve significantly decreased when MDA-MB-231 cells lack overexpressed MIEN1 protein implying that MIEN1 promotes actin polymerization. To further confirm that the observed cytoskeletal rearrangements were a direct consequence of MIEN1 depletion, GFP and GFP-tagged MIEN1 expressing MDA-MB-231 cells were treated with Cytochalasin D, an inhibitor of actin polymerization [36] for 1 h. As expected, Cytochalasin D caused disruption of the actin cytoskeleton in MDA-MB-231 cells expressing GFP whereas GFP-MIEN1 expressing cells treated with Cytochalasin D partially maintained their membrane integrity (Figure 4D). These results collectively suggest that MIEN1 induces membrane protrusion formation, stabilizes F-actin polymerization, and supports the transition of G-actin to F-actin.

### MIEN1-driven signaling and cytoskeletal changes are required for breast cancer cell motility

**Figure 5:**
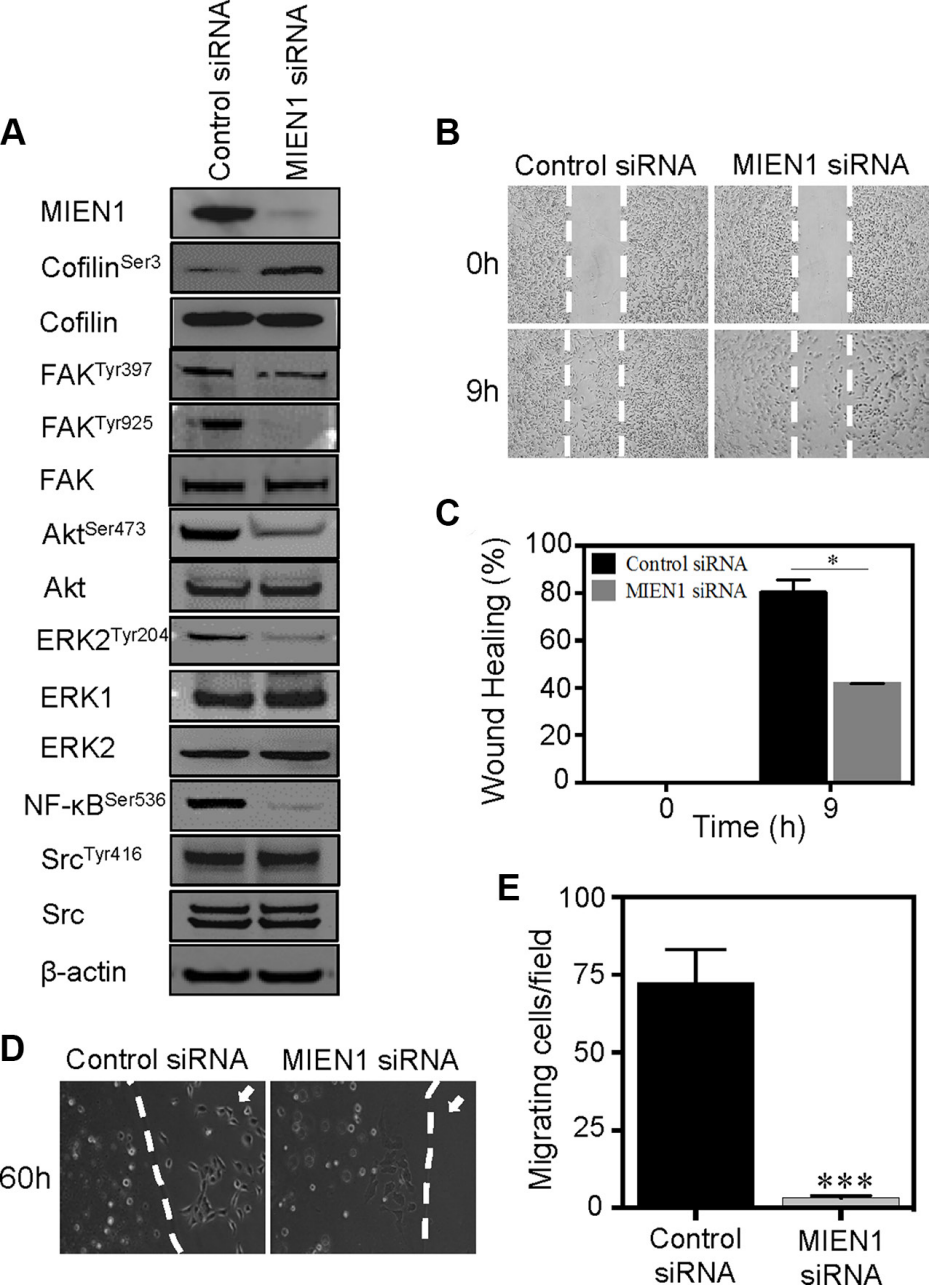
MIEN1 depletion inhibits the cell motility and associated signaling in breast cancer cells (**A**) MDA-MB-231 cells transfected with control or MIEN1 siRNA were collected at 72 h post-transfection. The cells were lysed and subjected to Western blot analysis with the indicated antibodies. (**B**) MDA-MB-231 cells were treated with control or MIEN1 siRNA for 72 h and subjected to wound healing upon sub-confluency. The wounded areas were photographed directly after the wounding and at the indicated time points. Representative images of the wound closure are shown. (**C**) Quantification of the wound-healing assays in MDA-MB-231 is depicted. (*p < 0.05) (**D**) Representative images of agarose bead assay in MDA-MB-231 cells treated with control or MIEN1 siRNAs. (**E**) Quantification of number of cells migrated out from at least 10 independent fields. (***p < 0.001 vs control).

To examine potential mechanisms by which MIEN1 contributes to cytoskeletal changes and adhesion, we analyzed the signaling pathways involved in cell motility. Cofilin, a key regulator of actin filament dynamics, binds to G- and F-actin to promote actin filament turnover [35, 37–40]. Based on our previous observations, we analyzed the actin dependent cofilin pathway to determine whether MIEN1 acts through this pathway to regulate cytoskeletal dynamics. We found that depletion of MIEN1 induced a larger pool of phosphorylated or inactive cofilin. Analysis of the phosphorylation status of cofilin at Ser- 3 revealed a 4-fold increase in cofilin phosphorylation in MIEN1 siRNA treated MDA-MB-231 and 5-fold increase in MIEN1 siRNA treated MCF10CA1a cells compared to control cells (Figure 5A and Supplementary Figure S3A). The total cofilin remained unchanged in both cell lines upon silencing of MIEN1. FAK is another key signaling molecule that regulates both focal adhesion and motility through phosphorylation and dephosphorylation of its tyrosine residues [32, 41]. Phosphorylation of FAK at Tyr-397 leads to the recruitment of Src family kinases and induction of several downstream signaling pathways, while phosphorylation at Tyr-925 regulates cross talk between focal adhesion turnover and cell protrusions [32, 41–43]. Interestingly, treatment of MDA-MB-231 and MCF10CA1a cells with MIEN1 siRNA led to a significant decrease of FAK phosphorylation at Tyr-925 but not Tyr-397 compared to the control siRNA treated cells (Figure 5A and Supplementary Figure S3A). There was no detectable alteration in total FAK expression. Taken together, these results indicated that MIEN1 exerts its effects on cell adhesion and motility through the cofilin and FAK pathways. In addition, MIEN1 knockdown also impaired the phosphorylation of other cell motility markers, including Akt on Ser-473, ERK1/2 on Thr-202/Tyr-204, NF-kB on Ser-536 but not Src on Tyr-416 in both cell types (Figure 5A and Supplementary Figure S3A). Similarly, the total levels of these proteins remained unaffected.

Since we know that cytoskeletal changes and signaling are key driving forces of cell motility and MIEN1 influences these processes, it is expected that MIEN1 knockdown will reduce overall cell motility. Using both wound-healing and chemotaxis-induced migration assays, we assessed the effects of MIEN1 knockdown on breast cancer cell migration. As shown in Figure 5B and 5C, depletion of MIEN1 reduced tumor cell migration in wound closure assay at 9 h compared to control siRNA treated MDA-MB-231 cells by almost 40%. The same effect was also observable in MCF10CA1a cells, where silencing of MIEN1 inhibited cell migration at both 9 and 18 h time intervals by approximately 15 and 25%, respectively (Supplementary Figure S3B and S3C). In chemotaxis-induced migration assays, we observed that control cells migrating out of the agarose bead toward the EGF-containing medium, quickly elongated and spread out (Figure 5D and 5E); while MIEN1 knockdown MDA-MB-231 cells exhibited significant reduction in motility. In addition, similar effects were also observed in MCF10CA1a cells, where knockdown of MIEN1 significantly reduced the cell motility in chemotaxis-induced migration assay (Supplementary Figure S3D and S3E). Collectively, our data suggest that MIEN1 is an essential signaling adaptor protein in breast cancer cells, which acts through various pathways to control actin rich protrusive structures and adhesion.

## DISCUSSION

Tumor cell migration is a key process for cancer cell dissemination and metastasis that is largely driven by dynamic reorganization of the actin cytoskeleton [2, 35, 44]. The procession of cell migration and invasion through the extracellular matrix begins with actin polymerization and protrusion of the cell membrane [3, 28, 32, 40]. Here, we demonstrate that MIEN1 acts as a cytoskeletal-signaling adapter protein to drive breast cancer cell migration. Predominantly a cytoplasmic and membrane protein, MIEN1 localized to the lamellum, a region known to play a major role in actin-rich membrane protrusions during cell migration. Thus, we investigated the effects of MIEN1 on actin-rich membrane protrusions. Our results demonstrate that modulation of MIEN1 expression leads to the re-organization of actin-rich membrane protrusions in breast cancer cells. Because membrane protrusions and cell adhesion are interconnected, we speculated that MIEN1 depletion might affect cell-substratum interactions. Consequently, down-regulation of MIEN1 significantly decreased cell adhesion and motility. These results are consistent with previous reports which attest to the role of MIEN1 in tumor cell motility and demonstrate for the first time that MIEN1 is also important for cell-substratum interactions.

At the leading edge of a migrating cell, actin filaments contribute to membrane protrusions and provide mechanical strength for cell adhesion. The growing actin filaments occur as a result of G-actin polymerizing into F-actin [20, 40]. Given our observations, we investigated if MIEN1 is involved in the assembly of actin filaments. Examination of F-actin and G-actin ratio showed a distinct shift toward the prevalence of F-actin in control cells compared to MIEN1 down-regulated cells suggesting MIEN1 might be required for filamentous actin assembly. Over-expression of MIEN1 led to an increase in actin polymerization. Furthermore, treatment of MIEN1 over-expressing cells with Cytochalasin D, which inhibits the binding of G- and F-actin to cofilin [36], demonstrated that MIEN1 is essential for G-actin to F-actin dynamics in breast cancer cells. Cofilin, a key regulator of actin filament dynamics, promotes actin polymerization and defines the direction of cell motility [45]. Accumulating evidence shows that both cancer invasion and metastasis are directly linked to activation of cofilin, which is regulated by phosphorylation status on Ser-3 [46]. Cofilin phosphorylated on Ser-3 is unable to bind actin and has been considered an inactive form [39, 45]. Here, we provide evidence that depletion of MIEN1 down-regulates cofilin activity by increasing its phosphorylation at Ser- 3, which then inhibits actin polymerization, membrane protrusion, and cell migration. These results further confirmed that cofilin activity is essential for the localized formation of barbed ends, which determine the direction of cell protrusion and movement [38, 47].

Focal adhesion kinase is an important regulator of cell motility and can affect the activation of various downstream signaling pathways [25, 27, 32, 41, 48, 49]. In breast tumors, FAK mediates tumor cell adhesion, and its overexpression induces survival signals to promote breast cancer growth and metastasis [42, 50, 51]. Furthermore, our results demonstrate that depletion of MIEN1 leads to a reduction in FAK phosphorylation at Tyr-925 along with reduced phosphorylation of previously shown markers of motility such as Akt, Erk1/2 and NF- κB [14, 18, 19, 43, 52]. These results are consistent with previous findings which demonstrate that Src-mediated phosphorylation of FAK at Tyr-925 creates a binding site for Grb2 and then stimulate mitogen-activated protein kinase (MAPK) signaling via a FAK/Grb2/Sos/Ras/Erk2 pathway [43, 52]. In contrast, depletion of MIEN1 did not affect the expression level of active phospho-Src (Tyr- 416). The mechanism through which MIEN1 regulates the phosphorylation of FAK at Tyr-925 still remains unknown. However, these observations further support our hypothesis that MIEN1 promotes cellular adhesion and the actin dynamics by inducing phosphorylation of FAK at Tyr-925 and reducing phosphorylation of cofilin at Ser-3, which results in breast cancer cell migration.

The finding that MIEN1 activates the cytoskeletal regulator cofilin and enhances actin polymerization during cell migration has not been reported previously. Our data reveals a novel mechanism whereby MIEN1 promotes actin reorganization and subsequently induces breast tumor cell migration through cofilin activation. In addition to providing evidence further establishing the role MIEN1 in cell migration, our studies demonstrate a novel role for MIEN1 in cell adhesion and signaling. Furthermore, we demonstrate that MIEN1 is a resident protein of the lamellum that is involved in F-actin assembly. The dissemination process critically depends on dynamic reorganization of the actin cytoskeleton. Gaining a better understanding of this process may help design new and more effective means to control invasion and ultimately distant metastases. Obtaining comprehensive metastatic gene signatures may eventually be important in the clinical management of breast cancer. Hence, validating the role of MIEN1 might indirectly be important in preventing breast tumor metastases.

## MATERIALS AND METHODS

### Antibodies and reagents

Antibodies used were: MIEN1 (Abnova, Taipei, Taiwan, and Life Technologies, Grand Island, NY), β-actin, ERK1, ERK2, ERK1/2, FAK, Paxillin, Tyr- 31 phosphorylated Paxillin (Santa Cruz Biotechnology, Dallas, TX), Ser-473 phosphorylated Akt, Akt, Tyr-416 phosphorylated Src, Src, Ser-3 phosphorylated Cofilin, Cofilin, Ser-536 phosphorylated NF-κB (Cell Signaling, Danvers, MA), Tyr-925 phosphorylated FAK (Life Technologies, Grand Island, NY), Tyr-397 phosphorylated FAK (Assay Biotech, Sunnyvale, CA). Alexa Fluor 488 phalloidin, Rhodamine phalloidin, Alexa Fluor 594 deoxyribonuclease I, Prolong Gold Antifade Mountant with DAPI were acquired from Molecular Probes (Life Technologies, Grand Island, NY). Human MIEN1 smart pool siRNA (L-014864-00) and control, non-targeting small interfering RNA pool (D-001810) were purchased from Dharmacon (Lafayette, CO).

### Molecular cloning

GFP-MIEN1^WT^ construction has been described previously [14]. Briefly, full-length human MIEN1 cDNA (347 base pairs) was amplified and directionally cloned into a pEGFP-C1 vector.

### Cell lines and culture conditions

Human breast epithelial cancer cell lines, MCF10CA1a and MDA-MB-231, were obtained from Barbara Ann Karmanos Cancer Institute (Detroit, MI) and American type culture collection (Manassas, VA), respectively. MCF10CA1a and MDA-MB-231 cells were cultured in DMEM/F12 or DMEM and supplemented with 10% fetal bovine serum or 5% horse serum, respectively. MCF10CA1a cell culture was further supplemented with hydrocortisone (0.5 mg/ml), cholera toxin (100 ng/ml), insulin (10 μg/ml) and EGF (20 ng/ml). All cell lines were maintained in a humidified incubator containing 5% CO_2_/95% air at 37°C.

### Transfections

MCF10CA1a and MDA-MB-231 cells were transfected with GFP vector control or GFP-tagged MIEN1 construct (GFP -MIEN1^WT^) using jetPRIME (VWR, Radnor, PA) as recommended by the manufacturer. Stable cells were selected with G418 (Life Technologies, Grand Island, NY). Gene knockdown experiments were carried out with control, non-targeting small interfering RNA pool and MIEN1 smart pool siRNA at 50 nM using Opti-MEM I Reduced Serum medium (Life Technologies, Grand Island, NY) in the presence of Lipofectamine RNAiMAX (Life Technologies, Grand Island, NY). Cells were incubated up to 72 h, validated for target protein knockdown by western blotting and processed for subsequent experiments.

### Cell migration and invasion assay

Breast tumor cells were grown on fibronectin (5 μg/ml) coated plates and transiently transfected with control, non-targeting smart pool and MIEN1 smart pool siRNAs. Confluent monolayers of cells were wounded using a 10 μl pipette tip. The wounded areas were photographed directly after wounding (0 h) and at regular interval up to 18 h using a fluorescence microscope (Olympus, Waltham, MA). A soft agar bead assay was used to confirm the migratory potential of the cells in the presence of MIEN1. Breast tumor cells transfected with control or MIEN1 targeting siRNA were trypsinized, counted and suspended in 1% low–melting point agarose solution to obtain a final concentration of 0.5%. 25 μl of the agar-cells suspension was dropped in each well of a 6-well plate pre-coated with fibronectin (5 μg/ml) to form beads. Following incubation of the beads at 4°C for 15 min, EGF supplemented media without serum was added. Following incubation of the plates, cell morphology and migration were observed at the specific times indicated. Three independent experiments were performed for each cell line in triplicate.

### Cell adhesion assay

siRNA treated breast tumor cells were trypsinized, washed in serum-free medium containing soybean trypsin inhibitor and pelleted. 1 × 10^6^ cells/ml were suspended in serum-free medium containing 0.5% bovine serum albumin (BSA) and 100 μl was added to fibronectin (5 μg/ml) coated coverslips. The cells were incubated at 37°C in 5% CO_2_/95% air atmosphere for the time periods specified in the results. At least 15 images were taken from random locations using phase contrast microscopy and the experiment was repeated at least twice.

### Immunofluorescence

Cells were grown on coverslips and fixed in 4% paraformaldehyde (Affymetrix, Santa Clara, CA) for 30 min at room temperature and washed with phosphate buffered saline (PBS). The cells were permeabilized in 0.2% Triton X-100 for 30 min at 4°C, blocked in 1% BSA for 1 h at room temperature and incubated with the primary antibodies overnight at 4°C. Secondary antibodies were added to fixed cells for 1 h at room temperature. PBS washes were performed following primary and secondary antibody incubation. Coverslips were mounted on microscopy slides using Prolong Gold Antifade Mountant with DAPI (Invitrogen Inc., Eugene, OR). Fluorescence images were acquired using LSM 510 META confocal system (Carl Zeiss, Thornwood, NY).

### Immunoblotting

Cells were lysed in NP-40 buffer containing protease and phosphatase inhibitors cocktail (EMD Millipore, Billerica, MA). Protein concentrations were determined by Pierce BCA protein assay kit (Thermo Scientific, Rockford, IL). Cell extracts were separated on 4–12% Bis-Tris NuPAGE gel (Life Technologies Corporation, Carlsbad, CA) using MES buffer and transferred onto nitrocellulose membrane. Membranes were blocked with 5% fat-free milk in Tris-buffered saline containing 0.05% Tween 20 (TBST) at room temperature for 60 min, and incubated overnight at 4°C with the appropriate primary antibody in 5% milk in TBST. After three washings with TBST, the membrane was incubated with the appropriate secondary antibody (SouthernBiotech, Birmingham, AL) at room temperature for 2 h. After washing again with TBST, the membranes were developed using Immobilon Western Chemiluminescent HRP substrate (Millipore Corporation, Billerica, MA), and the image was captured using alpha-imager Fluoretech HD2.

### Actin polymerization assays

*In vitro* actin polymerization assays were performed using the Actin polymerization Biochem kit (BK003, Cytoskeleton, Denver, CO) in which the rate of pyrene-labelled G-actin conversion into F-actin was monitored. Actin polymerization was measured in the presence of MDA-MB-231 cell lysates expressing GFP vector control or GFP-MIEN1 WT. Cell lysates with equal amount of total protein was added to the final reaction volume (200 μl). Pyrene fluorescence (λ_ex_ = 365 nm and λ_em_ = 407 nm) was recorded over time to monitor actin polymerization.

### Cytochalasin experiments

Stably transfected cells were treated with 1 μM Cytochalasin D (Cayman chemicals, Ann Harbor, MI) for 1 h. Cells were then collected following treatment and processed for further experimentation.

### Statistical analysis

The Student *t*-test was used for statistical analysis. All data are presented as the mean ± standard error of the mean (s.e.m.). A *P*-value of less than 0.05 was considered to be significant.

## SUPPLEMENTARY MATERIALS



## Abbreviations

EGF: Epidermal growth factor
EMT: Epithelial to mesenchymal transition
ERK: Extracellular-signal-regulated kinase
FAK: Focal Adhesion Kinase
F-actin: Filamentous-actin
G-actin: Globular-actin
GFP: Green fluorescent protein
GGTaseI: Geranylgeranyl transferase I
HER2/neu: Human epidermal growth factor receptor 2
ITAM: Immuoreceptor tyrosine-based activation motif
MMP-9: Matrix metallopeptidase 9
MIEN1: Migration and invasion enhancer 1
NF-κB: Nuclear factor kappa-light-chain-enhancer of activated B cells
siRNA: Short interfering RNA
uPA: Urokinase-type plasminogen activator
VEGF: Vascular endothelial growth factor
