# Supplementary material for: MIEN1 drives breast tumor cell migration by regulating cytoskeletal-focal adhesion dynamics
Source: Oncotarget. 2016 Jul 23;7(34):54913–24. doi: 10.18632/oncotarget.10798 (PMC5342390; doi:10.18632/oncotarget.10798)
Supplement: Supplementary file 1 [file oncotarget-07-54913-s001.pdf]

MIEN1 drives breast tumor cell migration by regulating cytoskeletal-focal adhesion dynamics

Supplementary Materials

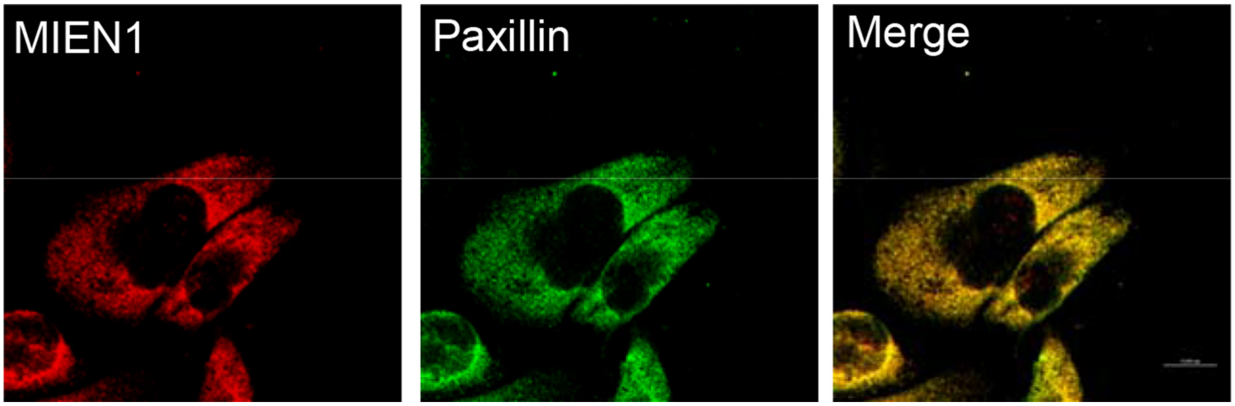

Supplementary Figure S1: Immunofluorescence staining of MIEN1 (Red) and paxillin (Green) in MCF10CA1a cells. Yellow dots represent the colocalization of green and red colors, which indicates that MIEN1 is colocalized with paxillin.

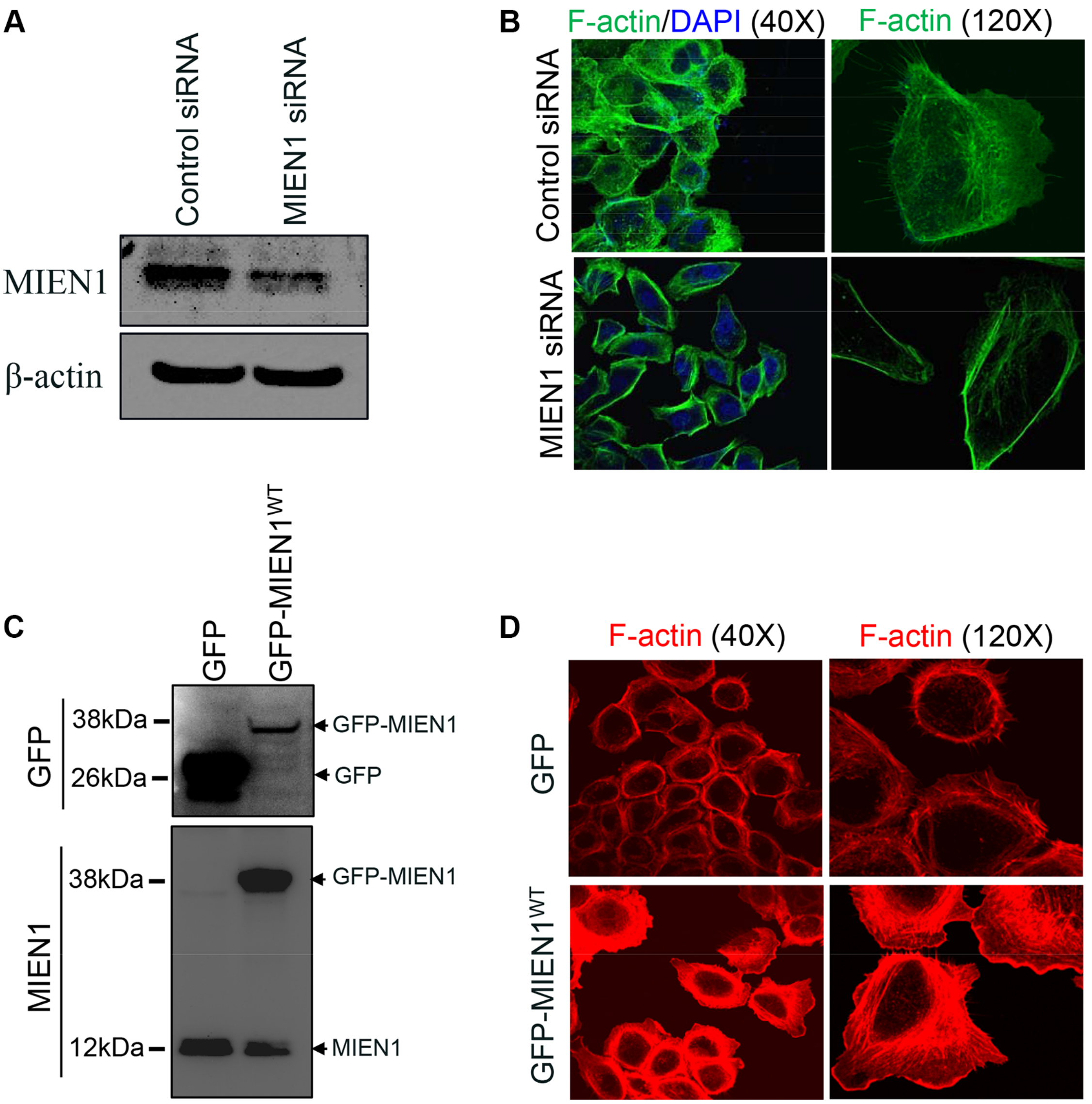

Supplementary Figure S2: MIEN1 regulates actin-rich membranes extensions during cell migration. (**A**) MCF10CA1a cells were treated with control or MIEN1 siRNA. Extracts were analyzed by western blotting at 72 h post-transfection. MIEN1 expression levels were significantly reduced in MIEN1 siRNA treated cells, whereas β-actin was unaffected. (**B**) Immunofluorescence analysis was performed using Alexa 488-labeled phalloidin (F-actin; green and DAPI; blue). (**C**) Western blot analysis shows MIEN1 expression upon GFP vector control or GFP-MIEN1 plasmid transfection in MCF10CA1a cells. (**D**) Cells expressing the GFP vector control or GFP-MIEN1 plasmid constructs were fixed and stained with rhodamine-conjugated phalloidin (F-actin) and examined for membrane protrusions.

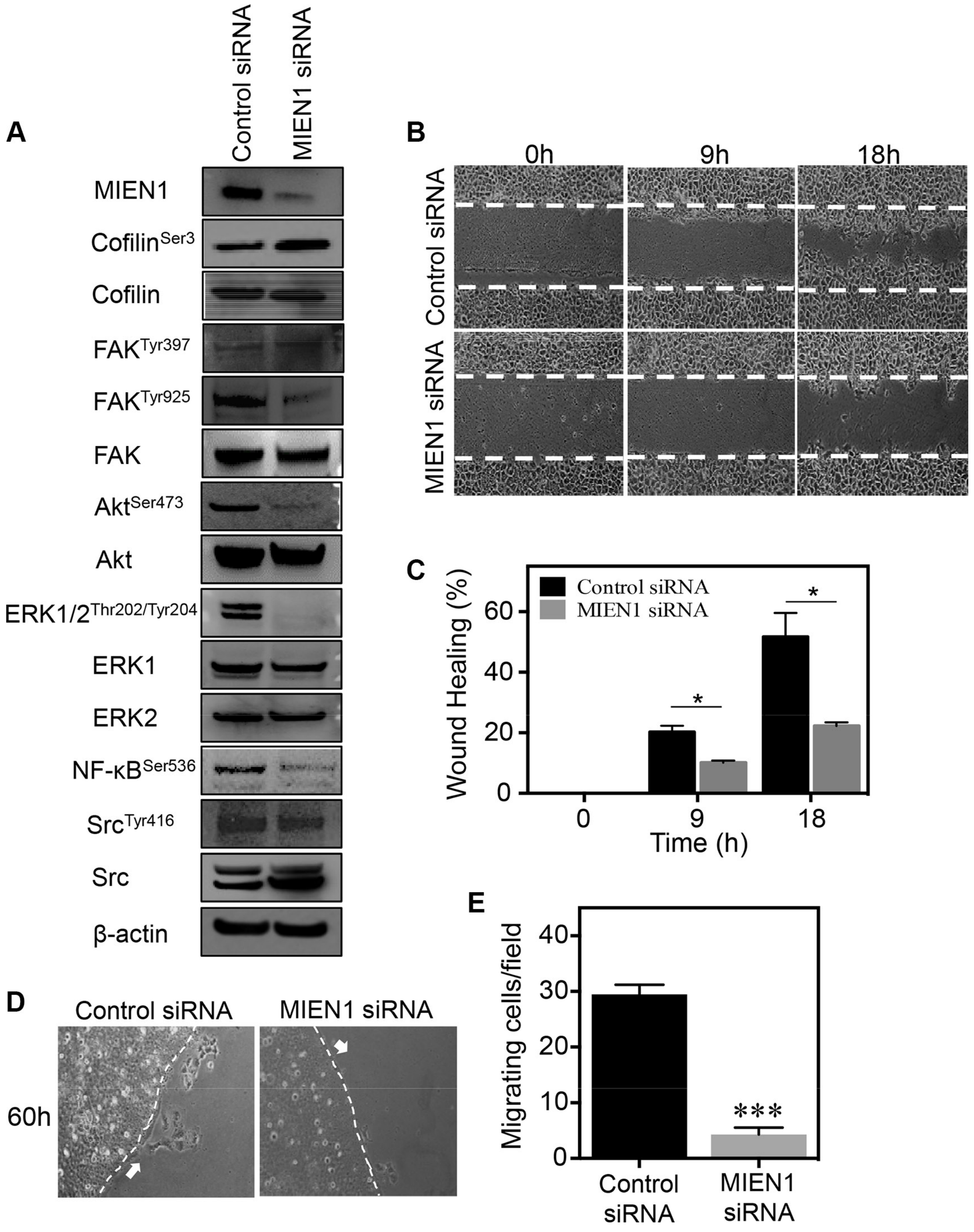

Supplementary Figure S3: MIEN1 depletion inhibits the breast cancer cells motility and associated signaling. (**A**) MCF10CA1a cells transfected with control or MIEN1 siRNA were collected at 72 h post transfection. The cells were lysed and subjected to Western blot analysis with the indicated antibodies. (**B**) MCF10CA1a cells were treated with siRNAs for 72 h and subjected to wound healing upon sub-confluency. The wounded areas were photographed at the indicated time points. Representative images of the wound closure are shown along with quantification (**C**). (**D**) Representative images of agarose bead assay along with quantification of number of cells migrating out of agarose beads (**E**). (*$P < 0.05$; ***$P < 0.001$ vs control).
